# What is the relative contribution of biological and psychosocial factors to the generation of hypoxia headache?

**DOI:** 10.1080/24740527.2018.1478224

**Published:** 2018-06-22

**Authors:** Diletta Barbiani, Eleonora Camerone, Fabrizio Benedetti

**Affiliations:** a Neuroscience Department, University of Turin Medical School, Turin, Italy; b Plateau Rosà Laboratories, Plateau Rosà, Italy/Switzerland; c Institute of Psychiatry, Psychology & Neuroscience, King’s College, London, UK

**Keywords:** headache, biopsychosocial model, hypobaric hypoxia, cyclooxygenase, prostaglandins, cortisol

## Abstract

**Background:**

The biopsychosocial model claims that illness is generated by both biological and psychosocial factors. Accordingly, several studies have shown that both factors contribute to the generation of pain.

**Aims:**

The aim of the present study is to manipulate biological, psychological, and social factors in hypobaric hypoxia headache in order to understand their relative contribution to the generation of headache pain.

**Methods:**

Healthy subjects were subdivided into three groups and brought to our high-altitude labs for the assessment of hypoxia-induced headache, blood oxygen saturation (SO_2_), prostaglandins, and cortisol during the first 24 h after arrival. The first group did not undergo any manipulation. The second group (negative expectation) was told that severe headache would occur if SO_2_ dropped to less than 80% and their oximeters were set to display a saturation of 75%, even though real SO_2_ was much higher. The third group (negative expectation and social interaction) underwent the same manipulation as the second group, but these subjects spent the night together with people experiencing headache and insomnia.

**Results:**

Although none of the three groups differed significantly for SO_2_, the second group, compared to the first, experienced more severe headache and showed an increase in prostaglandins and cortisol. The third group, compared to the second group, showed a further increase of headache as well as of prostaglandin (PG) E2 and cortisol.

**Conclusions:**

These findings indicate that biological, psychological, and social factors are additive not only in the generation of headache but also for the biochemical changes related to hypoxia.

## RÉSUMÉ

**Contexte** : Le modèle biopsychosocial affirme que la maladie est engendrée à la fois par des facteurs biologiques et psychosociaux. Plusieurs études ont donc démontré que ces deux types de facteurs contribuent à engendrer de la douleur.

**But** : Le but de la présente étude est de manipuler les facteurs biologiques, psychologiques et sociaux dans les cas de céphalées associées à l’hypoxie hypobarique afin de comprendre leur contribution relative dans l’apparition de la douleur occasionnée par les céphalées.

**Méthodes** : Des sujets en bonne santé ont été répartis en trois groupes et ont été conduits dans des laboratoires à haute altitude afin d’évaluer les céphalées induites par hypoxie, la saturation de l’oxygène dans le sang (SO2), les prostaglandines et le cortisol au cours des premières 24 heures suivant leur arrivée. Le premier groupe n’a été soumis à aucune manipulation. Le second groupe (attente négative) a été informé qu’une céphalée sévère surviendrait si le SO2 diminuait à moins de 80 % et les oxymètres des sujets de ce groupe ont été réglés de manière à indiquer une saturation de 75 %, même si le SO2 réel était beaucoup plus élevé. Le troisième groupe (attente négative et interaction sociale) a été soumis à la même manipulation que le second groupe, mais ces sujets ont passé la nuit avec des personnes souffrant de céphalées et d’insomnie.

**Résultats** : Bien qu’aucun de ces trois groupes ne différait de manière sinificative en ce qui a trait au SO2, le deuxième groupe, comparativement au premier, a souffert de céphalées plus sévères, en plus de démontrer une augmentation des prostaglandins et du cortisol. Le troisième groupe, comparativement au deuxième, a démontré une augmentation des céphalées, des prostaglandines (PG) E2 et du cortisol encore plus marquée.

**Conclusions** : Ces résultats indiquent que les facteurs biologiques, psychologiques et sociaux sont cumulatifs non seulement dans l’apparition de la céphalée, mais aussi dans les changements biochimiques liés à l’hypoxie.

## Introduction

In 1977, Engel^1^ challenged the medical and scientific community by putting forward a new medical model that takes into account biological, psychological, and social factors as important determinants of illness. According to this biopsychosocial model, the anatomy, physiology, and biochemistry of different organs and systems take an important part in the emergence and course of illness, yet they are not enough, because additional factors must be included in a global model of illness. Many psychological and social aspects have been recognized over the centuries by physicians and psychologists as contributing factors to the emergence of certain diseases, but the scientific formulation of such a contribution is relatively recent.^2^ This is attributable to the recent emergence of modern concepts in psychosomatics, psychoneuroimmunology, and psychoneuroendocrinology.

The basic idea of the biopsychosocial model is not so much to deny biomedical research but rather to criticize its narrow focus on the biochemical and physiological mechanisms.^1^ Indeed, emerging experimental evidence in modern medicine indicates powerful influences of the mind over the body, whereby the patient’s psychological state and the social factors impinging on him or her are all involved in both the pathophysiology and the treatment outcomes of a given disease.^3–6^ Recent research of placebo and nocebo effects support this model, whereby either positive or negative expectations about the therapeutic outcome may impact the response to a treatment.^7^ Indeed, placebo effects derive from the psychosocial context around the patient and the therapy.^7,8^ A positive context may lead to positive outcomes, or the placebo effect.^7–10^ Differently, a negative context may lead to negative outcomes, or the nocebo effect.^8,10–13^

Despite the general agreement in the scientific community about the interaction between biological mechanisms and psychosocial influences,^14^ these interactions are still little understood or completely unknown.^3–6^ Crucial unanswered questions include the following: Are biological, psychological, and social factors additive in the generation of illness? Are biological factors more important than psychosocial factors or vice versa? Can such interactions operate across a variety of illnesses or only in specific medical conditions? All of these questions are very much related to nocebo effects, which have been shown to be generated by negative expectations and whose biological underpinnings have been partially uncovered.^12,15–17^ Clearly, nocebo effects highlight the important interaction between biological and psychological factors in the generation of illness, particularly pain.^8,18^

In the present study, we use the model of hypobaric hypoxia, or high-altitude, headache to investigate the relative contribution of biological factors (hypoxia) and psychosocial factors (negative expectation and social interaction) in the generation of pain. To do this, we analyzed how headache pain, cyclooxygenase (COX) activity, cortisol, blood oxygen saturation (SO_2_), and heart rate (HR) were affected by hypoxia and by negative expectations and social interactions. This allowed us to understand the relative contribution of each of these elements in the generation of headache pain.

## Material and methods

### Subjects and study location

A total of 36 healthy subjects (18 males, 18 females), were recruited from the student population of the Medical School, Psychology School, and PhD Neuroscience Program of the University of Turin. The participants signed a written informed consent form in which the experimental procedure was described in detail after approval by the Ethics Committee of the School of Medicine. All subjects engaged in recreational fitness training and were asked to avoid hard exercise starting from at least 24 h before the experimental sessions. They were either nonsmokers or light smokers (less than 10 cigarettes/day). The subjects were randomly subdivided into three groups on the basis of a homemade computer randomization program, whose characteristics are summarized in Table 1. All subjects were asked to refrain from consuming coffee, tea, or other caffeine-containing beverages for 24 h before each experimental session, as well as alcohol and any drug.

**Table 1.** Subject characteristics in the three groups.^a^

	Group 1	Group 2	Group 3	*P* value
Number of subjects	12 (5 m)	12 (6 m)	12 (7 m)	0.71
Age (years)	29.33 ± 4.57	29.5 ± 4.01	28.91 ± 3.28	0.93
Weight (kg)	63.4 ± 10.23	68.33 ± 10.96	66.75 ± 12.22	0.55
BMI (kg/m^2^)	21.74 ± 3.12	22.29 ± 2.19	22.62 ± 2.39	0.87

^a^Values are means ± SD. m = males; BMI = body mass index.

All subjects went up to our laboratories at 3500 m through three gondolas, starting from an altitude of 2000 m in Breuil-Cervinia; all itineraries were exactly the same for all subjects. All experiments were performed under acute hypobaric hypoxia, that is, in the first 24 h after reaching 3500 m. The experiments were performed at the Plateau Rosà Laboratories in the Matterhorn area at the Italian–Swiss border, at an altitude of 3500 m. Here air pressure is 490 mmHg (760 mmHg at sea level) and oxygen pressure is 102 mmHg (159 mmHg at sea level). This corresponds to a blood oxygen saturation in the range of 85%–90% (98%–99% at sea level), depending on different individuals. Ambient temperature inside the laboratory was always maintained in a comfortable range (18–21°C).

### Experimental procedure

The subjects were subdivided into the following three groups.

Group 1 (natural history, *N* = 12; smokers = 5, nonsmokers = 7). These subjects left from Turin (altitude: 220 m) at 8:00 am on the first day. At 7:00 am, before leaving, SO_2_, HR, and headache “right now” and “last night” were assessed, the Lake Louise Score (LLS) questionnaire was administered, and saliva samples were taken. Arrival at our laboratories at 3500 m was around 11:00 am, and then they spent the whole day and the night at high altitude. At 7:00 am on the second day, the same measurements were taken (SO_2_, HR, headache “right now” and “last night”, LLS, saliva samples). This group represents a no-treatment group, in which we assessed the changes at high altitude compared to sea level.

Group 2 (negative expectation, *N* = 12; smokers = 6, nonsmokers = 6). The measurements were exactly the same as in group 1. However, these subjects were informed that they would experience severe headache if SO_2_ dropped below 80%. Indeed, their oximeter was manipulated, displaying a value of 75% for the whole day of their stay in our laboratories, even though SO_2_ was much higher than 80%. Therefore, the constant 75% value on the display of the oximeter made the subjects expect the occurrence of severe headache, also reported by the subjects themselves.

Group 3 (negative expectation and social interaction, *N* = 12; smokers = 7, nonsmokers = 5). This group underwent the same procedure and measurements as group 2. However, these subjects spent the night sharing a room with two skiers who were diagnosed with acute mountain sickness by means of LLS. Indeed, the two skiers complained of severe headache and insomnia, a very common situation in our high-altitude center. Therefore, the subjects in this group expected severe headache, due to their oximeter displaying a 75% SO_2_, and interacted with acute mountain sickness sufferers all night long, as also reported by the subjects themselves.

At the end of the study, all subjects were debriefed and the aim of the study was explained in detail. In particular, they were told that their oximeter was manipulated, displaying a value of 75%, even though their SO_2_ was higher than 80%. We did not observe any negative reaction and all subjects were enthusiastic about the study.

### Measurements and data analysis

Physiological parameters were monitored by means of an Equivital EQ02 LifeMonitor (Hidalgo, Cambridge, UK), providing recordings of the electrocardiogram and blood oxygen saturation (SO_2_). SO_2_ was also measured by means of a PM-60 pulse oximeter (Mindray, Shenzhen, China).

LLS questionnaire was administered to assess acute mountain sickness, whereby symptoms such as headache, dizziness, gastrointestinal symptoms, insomnia, and fatigue are evaluated by a doctor: each symptom is rated on a scale where 0 = *none*, 1 = *mild*, 2 = *moderate*, 3 = *severe*. Diagnosis of acute mountain sickness is made when the overall score is greater than 3. A score equal to or higher than 3 indicates the occurrence of acute mountain sickness. Headache was assessed by means of a numerical rating scale ranging from 0 = *no pain* to 10 = *unbearable pain*. This was done for “headache right now”, that is, in the morning (Pain M) and “headache last night” (Pain N).

Saliva samples were collected after stimulation with sterile 2% citric acid applied to the tip and sides of the tongue with a cotton-tipped applicator. To prevent mixing of stimulated and unstimulated saliva, the ﬁrst 2 min of saliva was discarded. Then citric acid was continually applied for an additional 2 min or until 2 mL of saliva was collected. Saliva was collected by means of a syringe. All samples were kept at −18°C until preparation for analysis. Analysis started by thawing the saliva samples at room temperature and recording the volume of each sample. Then, the samples were centrifuged at 3000 rpm at 4°C and the supernatant was utilized for prostaglandin (PG) and total protein analysis. We determined all of the main products of COX, the enzyme that transforms arachidonic acid into PG H2, which, in turn, is transformed into PGD2, PGE2, PGF2, PGI2, and thromboxane A2 (TXA2). Using enzyme-linked immunosorbent assay (ELISA) kits (Cayman Chemical, Ann Arbor, MI), we analyzed PGD2, PGE2, and 8-isoprostane PGF2a (PGF2) directly, whereas PGI2 (prostacyclin) was analyzed by assessing its stable metabolite 6-keto PGF1a, and TXA2 was assessed through its stable metabolite TXB2. In order to control for artifact variance in the ELISA assay, the amount of PG was normalized to the volume of saliva collected and amount of total protein, which was determined using a standard protein assay (Bio-Rad Laboratories, Hercules, CA).

Cortisol analysis was performed by thawing the saliva samples at room temperature and recording the volume of each sample. Then samples were centrifuged at 3000 rpm at 4°C. A salivary Cortisol ELISA Kit (Marburg, Germany) was used to measure salivary cortisol concentrations. The range of the assay was between 0.537 and 80 ng/mL, and the intra- and interassay variability coefficients were 1.5%–4.5% and 5.8%–7.5%, respectively.

### Statistical analysis

A first between-groups analysis was performed by means of one-way analysis of variance in order to see possible changes at baseline (sea level) across different groups. Gender balance was tested by means of the chi-square tests. A within-group analysis was performed by means of a paired *t* test to assess differences between sea level and high altitude. We also used Mauchly’s sphericity test to verify that the variances of the differences between all possible pairs of groups were equal. In no case was sphericity violated. Then, we performed a between-groups analysis by computing the differences of the means and the 95% confidence intervals (CIs). The expectation effect was expressed as the difference between natural history (group 1) and negative expectation (group 2), the social effect as the difference between negative expectation (group 2) and negative expectation/social interaction (group 3), and the global psychosocial effect as the difference between natural history (group 1) and negative expectation/social interaction (group 3).

## Results

A first between-groups analysis was performed in order to assess possible baseline differences at sea level across the three groups. Table 2 shows the means, CI, and levels of significance for all measurements performed at sea level. It is possible to see that there were no significant differences between the three groups, which indicates that the subjects of different groups started from the same baseline values.

Then we performed a within-group analysis. When the subjects were tested 24 h later at high altitude, group 1 showed an increase in all measurements, with the exception of PGD2 and cortisol (Table 3). Likewise, groups 2 and 3 showed an increase in all measurements when at high altitude, with the exception of PGD2 (Table 3). Therefore, whereas the natural history group did not show any increase in cortisol, the negative expectation and social groups showed a substantial increase in salivary cortisol, which indicates that cortisol increase is not attributable to high-altitude-induced hypoxia per se but rather to negative expectations.

**Table 2.** Means and CIs of the measurements at sea level in the three groups. No significant differences are present; thus, baseline values are the same across the three groups.

Outcome measures	Group 1	Group 2	Group 3	*P* value
LLS	0.66 (0.1 to 1.23)	0.33 (−0.08 to 0.74)	0.33 (−0.08 to 0.74)	0.45
Pain N	0	0.16 (−0.2 to 0.52)	0.16 (−0.08 to 0.4)	0.50
Pain M	0.08 (−0.09 to 0.25)	0	0	0.37
SO_2_	98.67 (98.38 to 98.95)	98.82 (98.61 to 99.02)	98.85 (98.57 to 99.12)	0.89
HR	64.23 (59.59 to 68.86)	61.89 (57.51 to 66.26)	64.2 (60.48 to 67.91)	0.81
PGD2	176.91 (162.58 to 191.23)	177.33 (163.16 to 191.49)	179.41 (163.46 to 195.35)	0.96
PGE2	4.3 (3.77 to 4.82)	4.2 (3.73 to 4.66)	3.98 (3.43 to 4.52)	0.86
PGF2	3.62 (3.2 to 4.03)	3.49 (3.02 to 3.95)	3.23 (2.86 to 3.59)	0.52
PGI2	14.87 (13.54 to 16.19)	14.43 (12.83 to 16.02)	14.97 (13.3 to 16.63)	0.94
TXA2	28 (26.01 to 29.98)	29.17 (27.44 to 30.89)	29.38 (27.71 to 31.04)	0.98
Cortisol	1.01 (0.86 to 1.15)	1.03 (0.89 to 1.16)	0.97 (0.88 to 1.05)	0.97

CI = confidence interval; LLS = Lake Louise Score; Pain N = headache last night; Pain M = headache right now (i.e., measured in the morning); SO_2_ = blood oxygen saturation; HR = heart rate; PG = prostaglandin; TXA2 = thromboxane A2.

**Table 3.** Mean (and CI) changes from sea level to high altitude in the three groups for all measurements performed.^a^

Outcome measures	^b^Significant changes	Sea level mean (CI)	High altitude mean (CI)	*P* value
**Group 1**				
**Natural history**				
LLS	*	0.66 (0.1 to 1.23)	3.33 (2.38 to 4.28)	<0.001
Pain N	*	0	2.75 (1.8 to 3.7)	<0.001
Pain M	*	0.08 (−0.09 to 0.25)	1 (0.33 to 1.66)	<0.02
SO_2_	*	98.67 (98.38 to 98.95)	88.96 (87.11 to 90.8)	<0.001
HR	*	64.23 (59.59 to 68.86)	85.27 (78.94 to 91.59)	<0.001
PGD2		176.91 (162.58 to 191.23)	198.91 (189.18 to 208.63)	0.077
PGE2	*	4.3 (3.77 to 4.82)	7.28 (6.53 to 8.02)	<0.002
PGF2	*	3.62 (3.2 to 4.03)	5.61 (5.22 to 5.99)	<0.005
PGI2	*	14.87 (13.54 to 16.19)	23.55 (21.66 to 25.43)	<0.002
TXA2	*	28 (26.01 to 29.98)	48.97 (47.11 to 50.82)	<0.001
Cortisol		1.01 (0.86 to 1.15)	1.1 (0.92 to 1.27)	0.36
**Group 2**				
**Negative**				
**expectation**				
LLS	*	0.33 (−0.08 to 0.74)	4.5 (3.58 to 5.41)	<0.001
Pain N	*	0.16 (−0.2 to 0.52)	3.91 (2.95 to 4.86)	<0.001
Pain M	*	0	2.16 (1.23 to 3.08)	<0.002
SO_2_	*	98.82 (98.61 to 99.02)	88.16 (87.05 to 89.26)	<0.004
HR	*	61.89 (57.51 to 66.26)	81.53 (76.84 to 86.21)	<0.01
PGD2		177.33 (163.16 to 191.49)	201.25 (190.68 to 211.81)	0.085
PGE2	*	4.2 (3.73 to 4.66)	11.32 (10.58 to 12.05)	<0.001
PGF2	*	3.49 (3.02 to 3.95)	10.5 (9.69 to 11.03)	<0.001
PGI2	*	14.43 (12.83 to 16.02)	27.5 (26.11 to 28.88)	<0.001
TXA2	*	29.17 (27.44 to 30.89)	62.33 (58.8 to 65.85)	<0.001
Cortisol	*	1.03 (0.89 to 1.16)	1.78 (1.59 to 1.96)	<0.01
**Group 3**				
**Negative expectation**				
**+ negative social interaction**				
LLS	*	0.33 (−0.08 to 0.74)	5.5 (4.86 to 6.13)	<0.001
Pain N	*	0.16 (−0.08 to 0.4)	5.66 (4.98 to 6.33)	<0.001
Pain M	*	0	3.58 (2.74 to 4.41)	<0.001
SO_2_	*	98.85 (98.57 to 99.12)	88.02 (86.79 to 89.24)	<0.002
HR	*	64.2 (60.48 to 67.91)	83.6 (80.84 to 86.35)	<0.01
PGD2		179.41 (163.46 to 195.35)	203.25 (192.92 to 213.57)	0.082
PGE2	*	3.98 (3.43 to 4.52)	13.05 (12.18 to 13.91)	<0.001
PGF2	*	3.23 (2.86 to 3.59)	10.3 (9.6 to 10.99)	<0.001
PGI2	*	14.97 (13.3 to 16.63)	28.01 (26.62 to 29.39)	<0.001
TXA2	*	29.38 (27.71 to 31.04)	61.04 (56.86 to 65.21)	<0.001
Cortisol	*	0.97 (0.88 to 1.05)	2.6 (2.33 to 2.86)	<0.005

^a^LLS values > 3 indicate acute mountain sickness. Pain SO_2_ values are expressed as %, HR as beats/min, PG and TX as nmol/mg, cortisol as μg/dL. ^b^Significant changes are shown with an asterisk. CI = confidence interval; LLS = Lake Louise Score; Pain N = headache last night; Pain M = headache right now (i.e., measured in the morning); SO_2_ = blood oxygen saturation; HR = heart rate; PG = prostaglandin; TXA2 = thromboxane A2.

**Figure 1.**
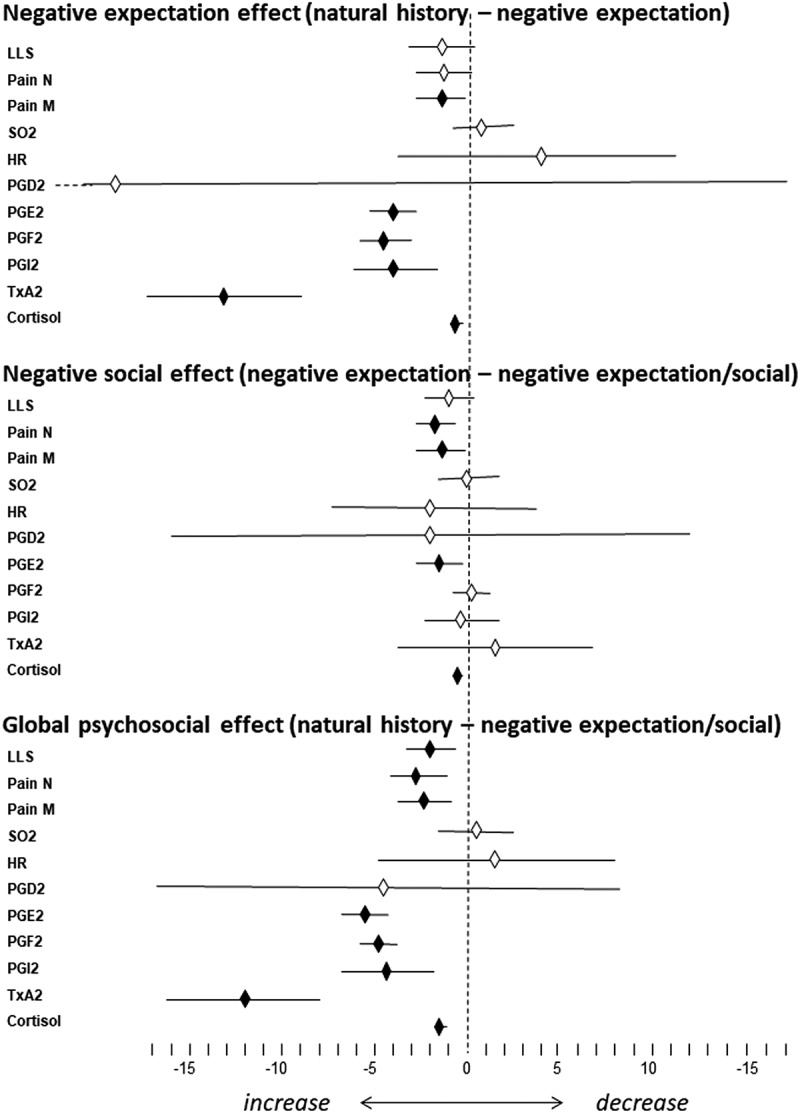
Differences in means and CIs between natural history and negative expectations (expectation effect), between negative expectation and negative social interaction (social effect), and between natural history and negative expectation/social (global psychosocial effect). CIs (horizontal bars) crossing the midline are not significant. Black diamonds = significant differences. White diamonds = nonsignificant differences.

In order to assess the relative contribution of biological, psychological, and social factors in the changes observed at high altitude, a between-groups analysis was carried out by calculating the differences of the means (Table 4 and Figure 1). It can be seen that the effect of negative expectations, calculated as the difference between natural history (group 1) and negative expectation (group 2), was significant for pain “right now” (in the morning), PGE2, PGF2, PGI2, TXA2, and cortisol. Thus, all of these parameters were enhanced by negative expectation compared to the biological factor alone (hypoxia). The effect of negative social interaction, calculated as the difference between negative expectation (group 2) and negative social interaction (group 3), was significant for pain at night, pain in the morning, PGE2, and cortisol. Therefore, these parameters were further increased by negative social interaction compared to negative expectation alone.

**Table 4.** Differences in means and CIs for all measurements performed at high altitude.^a^

**The effect of negative expectation**		
**Group 1 (natural history) − group 2 (expectation)**		
LLS		−1.17 (−2.41 to 0.07)
Pain N		−1.16 (−2.42 to 0.1)
Pain M	*	−1.16 (−2.23 to −0.09)
SO_2_		0.8 (−1.23 to 2.83)
HR		3.74 (−3.67 to 11.15)
PGD2		−19.14 (−55.44 to 17.16)
PGE2	*	−4.04 (−5.03 to −3.05)
PGF2	*	−4.89 (−5.73 to −4.05)
PGI2	*	−3.95 (−6.15 to −1.75)
TXA2	*	−13.36 (−17.11 to −9.61)
Cortisol	*	−0.68 (−0.92 to −0.44)
**The effect of negative social interaction**		
**Group 2 (expectation) − group 3 (social)**		
LLS		−1 (−2.05 to 0.05)
Pain N	*	−1.75 (−2.85 to −0.65)
Pain M	*	−1.42 (−2.59 to −0.25)
SO_2_		−0.14 (−1.42 to 1.7)
HR		−2.07 (−7.19 to 3.05)
PGD2		−2 (−15.92 to 11.92)
PGE2	*	−1.73 (−2.8 to −0.66)
PGF2		0.2 (−0.8 to 1.2)
PGI2		−0.51 (−2.36 to 1.34)
TXA2		1.29 (−3.86 to 6.44)
Cortisol	*	−0.82 (−1.12 to −0.52)
**The global effect of negative expectation and social interaction**		
**Group 1 (natural history) − group 3 (expectation + social)**		
LLS	*	−2.17 (−3.24 to −1.1)
Pain N	*	−2.91 (−4 to −1.82)
Pain M	*	−2.58 (−3.58 to −1.58)
SO_2_		0.94 (−1.15 to 3.03)
HR		1.67 (−4.83 to 8.17)
PGD2		−4.34 (−16.93 to 8.25)
PGE2	*	−5.77 (−6.85 to −4.69)
PGF2	*	−4.69 (−5.43 to −3.95)
PGI2	*	−4.46 (−6.66 to −2.26)
TXA2	*	−12.07 (−16.37 to −7.77)
Cortisol	*	−1.5 (−1.8 to −1.2)

^a^Significant changes are shown with an asterisk. CI = confidence interval; LLS = Lake Louise Score; Pain N = headache last night; Pain M = headache right now (i.e., measured in the morning); SO_2_ = blood oxygen saturation; HR = heart rate; PG = prostaglandin; TXA2 = thromboxane A2.

Overall, the global effect of negative expectation and negative social interaction, compared to the biological factor alone (natural history of hypoxia effect), was significant for LLS, pain at night, pain in the morning, PGE2, PGF2, PGI2, TXA2, and cortisol (Table 4 and Figure 1). Therefore, negative expectation and social interaction were crucial in the generation of both severe headache pain and the associated biochemical changes, with the exception of SO_2_, HR, and PGD2.

## Discussion

The main finding of the present study is that the biological factor (hypobaric hypoxia) is only partially responsible for the changes we observed at high altitude. Negative psychosocial factors were found to be crucial in both the generation of symptoms and the biochemical changes associated with hypoxia. In fact, whereas hypoxia alone not surprisingly generated acute mountain sickness and associated changes (Table 3), both negative expectation and negative social interaction worsened both symptoms and biochemical changes (Table 4 and Figure 1). The fact that the outcomes for LLS differed from Pain N/M is not surprising, because LLS evaluates not only headache but nausea/vomiting, dizziness, insomnia, and fatigue. For example, there were significant differences between groups 2 and 3 for Pain N/M but not for LLS.

Interestingly, most of the changes we observed in hypoxic conditions were additive. Negative expectation exacerbated hypoxia-induced changes and negative social interaction further exacerbated negative expectation-induced changes. These findings are compatible with the biopsychosocial model of illness proposed by Engel,^1^ whereby, in addition to the physiology and biochemistry of different organs and systems, psychosocial factors must be included in a global model of illness. For example, biological factors include genetics and environmental influences, whereas psychological factors include personality styles, coping behaviors, health beliefs, and social factors such as doctor–patient communication, social class, and observation of others’ behaviors.^1–8^ Indeed, in our study, we considered hypoxia as a biological environmental influence, negative expectation about SO_2_ < 80% as a health belief, and negative social contact as the observation of others’ behaviors. The global experience of hypobaric hypoxia headache derived from a complex interplay between different elements, such as negative beliefs and negative social contacts. This may have important implications not only for the pathophysiology of headache but for its treatment as well.^19,20^ For example, our study clearly shows that reducing negative expectations and negative social contacts can be quite effective in mitigating the effects of hypoxia.

Recent research on placebo and nocebo has shown that the psychosocial context around the patient and the therapy may have an important impact on the therapeutic outcome.^8,18^ In particular, and related to the present study, nocebo effects have been shown to affect both the clinical symptoms and the associated biochemical and physiological changes of high-altitude hypoxia.^15^ For example, placebo and nocebo effects have been found to be associated to the modulation of COX activity in hypoxic conditions as well as to respiratory and cardiovascular activity.^15,18,21,22^ The present study was aimed at disentangling biological, psychological, and social factors in the generation of headache pain and in the modulation of COX activity. In this regard, the manipulations we used in this study to modulate expectations and social observation may be considered nocebo-like effects, whereby negative expectations and social contacts are crucial elements.

Some limitations of this study must be acknowledged. First, the sample of the subjects is relatively small, which is justified by the inherent difficulty of bringing healthy volunteers to high altitude. Thus, these findings will require further investigation with larger groups. Second, the biopsychosocial approach we used is limited to the model of hypobaric hypoxia headache, which is not necessarily true for other types of headache or pain or illness. Third, we did not measure other physiological parameters such as ventilation, blood pH, fatigue, or performance, as done in a previous study.^21^ Therefore, it will be interesting to investigate the overall performance—for example, fatigue tolerance—in future studies. Fourth, in the present study, it is difficult to understand the very nature of the social interaction between the experimental subjects and the two sick skiers. Indeed, they spent the night together and interacted with each other and the experimental subjects observed the administration of the LLS questionnaire and the diagnosis of acute mountain sickness. However, we cannot identify which factor was more important in the negative social interaction. An interesting challenge for future research will be to understand the role of different social factors—for example, observation and/or verbal interaction—in these negative social effects.

Surprisingly, only a few studies have considered the biopsychosocial model within the context of headache and migraine.^23–27^ We believe that our model of hypobaric hypoxia headache may help answer many unanswered questions as well as understand some of the interactions between biological and psychosocial factors. In fact, high-altitude headache is at the borderline between the clinical and the experimental setting, because it can be considered as a real clinical condition triggered by hypobaric hypoxia but, at the same time, it can be induced at will by simply bringing healthy volunteers to high altitude.^22^ In other words, it is a kind of clinical condition that can be induced experimentally. High-altitude headache is part of a clinical condition known as acute mountain sickness, which is usually diagnosed by means of the LLS questionnaire.^28^ This is aimed at detecting several symptoms, such as headache, nausea/vomiting, dizziness, insomnia, and fatigue, as well as neurological symptoms, which emphasizes the complex nature of this hypoxia-related clinical syndrome.

The hypoxia headache model is interesting for at least two reasons. On the one hand, acute mountain sickness is triggered by the drop in atmospheric oxygen pressure at high altitude.^29,30^ One important factor triggering high-altitude headache is represented by the acute effects of hypoxia on PG synthesis through the COX enzyme, with the formation first of PGH2 and then of PGF2, PGD2, PGE2, PGI2, and TXA.2.^15,31–35^ One of the most important effects of these eicosanoids is represented by vasodilation, which is thought to be the principal factor inducing acute hypoxia headache,^36–40^ although the direct stimulation of nociceptive afferents may also occur.^41^ On the other hand, high-altitude headache is modulated by both nocebo and placebo, along with a variety of biochemical parameters such as PG and TX, thus representing an excellent model to better understand the influences of psychological factors.^9–11^

In conclusion, hypobaric hypoxia headache is amenable to the experimental manipulation of both biological and psychosocial factors, yet is a clinical condition in all respects. We believe that further investigation of this condition will shed light on the complex interplay between biological and psychosocial factors in the generation of headache pain.

## References

[1] Engel G. The need for a new medical model: a challenge for biomedicine. Science. 1977;196:129–136. doi:10.1126/science.847460. 847460

[2] Moseley GL, Butler DS. Fifteen years of explaining pain—the past, present and future. J Pain. 2015;16:807–813. doi:10.1016/j.jpain.2015.05.005. 26051220

[3] Astin JA, Shapiro SL, Eisenberg DM, Forys KL. Mind–body medicine: state of the science, implications for practice. J Am Board Fam Pract. 2003;16:131–147. doi:10.3122/jabfm.16.2.131. 12665179

[4] Campbell CM, Edwards RR. Mind–body interactions in pain: the neurophysiology of anxious and catastrophic pain-related thoughts. Transl Res. 2009;153:97–101. doi:10.1016/j.trsl.2008.12.002. 19218091 PMC2738609

[5] Ray O. How the mind hurts and heals the body. Am Psychologist. 2004;76:288–294. 10.1037/0003-066X.59.1.29 14736318

[6] Van Oudenhove L, Cuypers S. The relevance of the philosophical “mind–body problem” for the status of psychosomatic medicine: a conceptual analysis of the biopsychosocial model. Medicine, Health Care and Philosophy. 2014;17:201–213. doi:10.1007/s11019-013-9521-1. 24443097

[7] Benedetti F. How the doctor’s words affect the patient’s brain. Eval Health Prof. 2002;25:369–386. doi:10.1177/0163278702238051. 12449081

[8] Benedetti F. Placebo and the new physiology of the doctor–patient relationship. Physiol Rev. 2013;93:1207–1246. doi:10.1152/physrev.00043.2012. 23899563 PMC3962549

[9] Peerdeman KJ, Van Laarhoven AIM, Keij SM, Vase L, Rovers MM, Peters ML, Evers AW. Relieving patients’ pain with expectation interventions : a meta-analysis. Pain. 2016;157:1179–1191. doi:10.1097/j.pain.0000000000000540. 26945235

[10] Tracey I. Getting the pain you expect : mechanisms of placebo, nocebo and reappraisal effects in humans. Nat Med. 2010;16:1277–1283. doi:10.1038/nm.2229. 20948533

[11] Benedetti F, Amanzio M, Casadio C, Oliaro A, Maggi G. Blockade of nocebo hyperalgesia by the cholecystokinin antagonist proglumide. Pain. 1997;71:135–140. doi:10.1016/S0304-3959(97)03346-0. 9211474

[12] Benedetti F, Amanzio M, Vighetti S, Asteggiano G. The biochemical and neuroendocrine bases of the hyperalgesic nocebo effect. J Neurosci. 2006;26:12014–12022. doi:10.1523/JNEUROSCI.2947-06.2006. 17108175 PMC6674855

[13] Colloca L, Finniss D. Nocebo effects, patient–clinician communication, and therapeutic outcomes. JAMA. 2012;307:567–568. doi:10.1001/jama.2012.115. 22318275 PMC6909539

[14] Mogil JS. Social modulation of and by pain in humans and rodents. Pain. 2015;156:S35–S41. doi:10.1097/01.j.pain.0000460341.62094.77. 25789435

[15] Benedetti F, Durando J, Vighetti S. Nocebo and placebo modulation of hypobaric hypoxia headache involves the cyclooxygenase–prostaglandins pathway. Pain. 2014;155:921–928. doi:10.1016/j.pain.2014.01.016. 24462931

[16] Colloca L. Nocebo effects can make you feel pain. Science. 2017;358:44. doi:10.1126/science.aap8488. 28983038 PMC5754642

[17] Tinnermann A, Geuter S, Sprenger C, Finsterbusch J, Büchel C. Interactions between brain and spinal cord mediate value effects in nocebo hyperalgesia. Science. 2017;358:105–108. doi:10.1126/science.aan1221. 28983051

[18] Benedetti F. Placebo effects: from the neurobiological paradigm to translational implications. Neuron. 2014;84:623–637. doi:10.1016/j.neuron.2014.10.023. 25442940

[19] Gordon A. The five pillars of pain management. Pain Manag. 2012;2:335–344. doi:10.2217/pmt.12.31. 24654719

[20] Keefe FJ, Porter L, Somers T, Shelby R, Wren AV. Psychosocial interventions for managing pain in older adults : outcomes and clinical implications. Br J Anaest. 2013;111:89–94. doi:10.1093/bja/aet129. PMC3690316 23794650

[21] Benedetti F, Dogue S. Different placebos, different mechanisms, different outcomes : lessons for clinical trials. PLoS ONE. 2015;10:e0140967. doi:10.1371/journal.pone.0140967. 26536471 PMC4633056

[22] Benedetti F, Durando J, Giudetti L, Pampallona A, Vighetti S. High-altitude headache : the effects of real vs sham oxygen administration. Pain. 2015;2:2326–2336. doi:10.1097/j.pain.0000000000000288. 26164587

[23] Andrasik F, Flor H, Turk D. An expanded view of psychological aspects in head pain : the biopsychosocial model. Neurol Sci. 2005;26:S87–S91. doi:10.1007/s10072-005-0416-7. 15926029

[24] Kröner-Herwig B, Morris L, Heinrich M. Biopsychosocial correlates of headache : what predicts pediatric headache occurrence? Headache. 2007;48:529–544. doi:10.1111/j.1526-4610.2007.00945.x. 18042227

[25] Leonardi M, Raggi A, Grazzi L, Amico DD. Disability, ICF biopsychosocial model and burden of migraine. J Headache Pain. 2015;16:6–7. doi:10.1186/1129-2377-16-S1-A2. 28132386 PMC4715013

[26] Martin PR, Forsyth MR, Reece J. Cognitive-behavioral therapy versus temporal pulse amplitude biofeedback training for recurrent headache. Behav Ther. 2007;38:350–363. doi:10.1016/j.beth.2006.10.004. 18021950

[27] Nicholson RA, Houle TT, Rhudy JL, Norton PJ. Psychological risk factors in headache. Headache. 2007;47:413–426. 17371358 10.1111/j.1526-4610.2006.00716.x PMC2408884

[28] Sutton JP, Coates G, Houston CS, Hackett PH, Oelz O. Hypoxia and mountain medicine. In: Sutton JR, Coates G, Houston CS, editors. Hypoxia and mountain medicine. Burlington, VT: Queen City Printers; 1992. p. 327–330.

[29] Imray C, Wright A, Subudhi A, Roach R. Acute mountain sickness : pathophysiology, prevention, and treatment. Prog Cardiovasc Dis. 2010;52:467–484. doi:10.1016/j.pcad.2010.02.003. 20417340

[30] Wilson MH, Newman S, Imray CH. The cerebral eff ects of ascent to high altitudes. Lancet Neurol. 2009;8:157–191. doi:10.1016/S1474-4422(09)70014-6. 19161909

[31] Amano T, Meyer JS. Prostaglandin inhibition and cerebrovascular control in patients with headache. Headache. 1982;22:52–59. doi:10.1111/hed.1982.22.issue-2. 7045037

[32] Richalet JI, Hornych A, Rathat C, Aumont J, Larmignat P. Plasma prostaglandins, leukotrienes and thromboxane in acute high altitude hypoxia. Resp Physiol. 1991;85:205–215. doi:10.1016/0034-5687(91)90062-N. 1947460

[33] Tassorelli C, Greco R, Armentero M, Blandini F, Sandrini G, Nappi G. A role for brain cyclooxygenase-2 and prostaglandine-e2 in migraine: effects of nitroglycerin. Int Rev Neurobiol. 2007;82:373–382. 17678972 10.1016/S0074-7742(07)82020-4

[34] Tuca JO, Planas JM, Parellada RP. Increase in PGE2 and TXA2 in the saliva of common migraine patients. Action of calcium channel blockers. Headache. 1989;29:498–501. doi:10.1111/hed.1989.29.issue-8. 2793453

[35] Wienecke T, Olesen J, Oturai PS, Ashina M. Prostaglandin E2 (PGE2) induces headache in healthy subjects. Cephalagia. 2009;29:509–519. doi:10.1111/j.1468-2982.2008.01748.x. 19187340

[36] Busse R, Förstermann U, Matsuda H, Pohl U. The role of prostaglandins in the endothelium-mediated vasodilatory response to hypoxia. Pflügers Arch Eur J Physiol. 1984;401:77–83. doi:10.1007/BF00581536. 6382148

[37] Davis RJ, Murdoch CE, Ali M, Purbrick S, Ravid R, Gordon S, Coleman RA. EP 4 prostanoid receptor-mediated vasodilatation of human middle cerebral arteries. Br J Pharmacol. 2004;141:580–585. doi:10.1038/sj.bjp.0705645. 14744815 PMC1574229

[38] Fredricks KT, Liu Y, Rusch NJ, Lombard JH. Role of endothelium and arterial K+ channels in mediating hypoxic dilation of middle cerebral arteries. Am J Physiol. 1994;267:H580–H586. 8067414 10.1152/ajpheart.1994.267.2.H580

[39] Messina EJ, Sun D, Koller A, Wolin MS, Kaley G. Role of endothelium-derived prostaglandins in hypoxia-elicited arteriolar dilation in rat skeletal muscle. Circul Res. 1992;71:790–797. doi:10.1161/01.RES.71.4.790. 1516156

[40] Ray CJ, Abbas MR, Coney AM, Marshall JM. Interactions of adenosine, prostaglandins and nitric oxide in hypoxia-induced vasodilatation : in vivo and in vitro studies. J Physiol. 2002;544:195–209. doi:10.1113/jphysiol.2002.023440. 12356892 PMC2290577

[41] Kawabata A. Lipid mediators and pain signaling: prostaglandin E2 and pain—an update. Biol Pharm Bull. 2011;34:1170–1173. doi:10.1248/bpb.34.1170. 21804197

